# Ethiodized poppyseed oil versus ioversol for image quality and adverse events in hysterosalpingography: a prospective cohort study

**DOI:** 10.1186/s12880-019-0346-0

**Published:** 2019-06-24

**Authors:** Yiqing Tan, Shilin Zheng, Wenfeng Lei, Fuhua Wang, Shengpan Jiang, Ting Zeng, Bei Zhou, Fan Hong

**Affiliations:** Department of Radiology, Wuhan Third Hospital, Tongren Hospital of Wuhan University, 241 Liuyang Road, Wuhan, 430063 Hubei China

**Keywords:** Hysterosalpingography, Ethiodized poppyseed oil, Ioversol, Image quality, Adverse events

## Abstract

**Background:**

This study aimed to investigate the image quality and adverse events (AEs) of ethiodized poppyseed oil (EPO) compared with ioversol as contrast agents in hysterosalpingography (HSG).

**Methods:**

Two hundred twenty-eight patients underwent HSG were consecutively recruited in this prospective cohort study, and were accordingly divided into EPO group (*N* = 165) and ioversol group (*N* = 63). The quality of image was assessed according to the European Guidelines on quality criteria for diagnostic radiographic images. AEs during, within 2 h and at 1-month post-HSG were recorded.

**Results:**

EPO displayed elevated image quality compared with ioversol including the total image quality score (*P* < 0.001), the cervical canal display score (*P* < 0.001), shape and outline of uterus score (*P* < 0.01), cervical mucosa or folds score (*P* < 0.001), oviduct isthmus score (*P* < 0.001), ampulla and fimbriae of oviduct score (*P* < 0.001) and celiac diffuse image score (*P* < 0.001). Multivariate linear regression displayed that EPO (*P* < 0.001) was an independent predictive factor for increased total image quality score. AEs were similar between EPO group and ioversol group during and within 2 h post-HSG (all *P* > 0.05). However, at 1-month post-HSG, the number of patients had unchanged and faded menstrual blood color decreased but the proportion of patients with deepened menstrual color increased in EPO group compared with ioversol group (*P* = 0.007). In addition, the number of patients had iodine residue in uterine cavity was elevated in EPO group compared with ioversol group (*P* < 0.001).

**Conclusion:**

EPO is more efficient in image quality and equally tolerant compared to ioversol as contrast agents in HSG.

**Electronic supplementary material:**

The online version of this article (10.1186/s12880-019-0346-0) contains supplementary material, which is available to authorized users.

## Background

Hysterosalpingography (HSG), using a contrast agent guided by computerized tomography (CT) in detecting tubal anomaly, is a diagnostic modality for patients with infertility, unusual uterine bleeding, congenital tubal malformation or tumor [1–3]. Among all the indications of HSG, infertility, as a growing problem defined as the absence of pregnancy beyond 1 year of unprotected intercourse, is perplexing millions of couples not only in developed countries but also in developing countries [4, 5]. As the leading cause of infertility, tubal-related disease, including tubal obstruction or occlusion, pelvic inflammatory disease, induced surgical abortion or ectopic pregnancy, etc., make infertility the most common indication for HSG [6–9].

Previous clinical experiences and studies suggest that HSG, in addition to its diagnostic property, is also capable of eliminating tubal obstruction and escalating the fertility rate [10]. The efficacy of HSG is influenced by choice of contrast agents, and clinical trials indicate that an oil-based contrast agent might have a better effect of enhancing the fertility rate post-HSG compared with water-soluble agent or no intervention [10, 11]. However, most previous studies focus on evaluating the safety profile and the improvement of fertility rate of oil-based versus water-soluble contrast agents, the efficiency concerning image quality are rarely compared between these two different types of contrast agents.

Therefore, the aim of our study was to investigate the image quality and adverse events (AEs) of ethiodized poppyseed oil (EPO) compared with ioversol as contrast agents in HSG.

## Methods

### Patients

A total of 228 patients underwent hysterosalpingography (HSG) between May 2017 and December 2017 at Department of Radiology, Tongren Hospital of Wuhan University were consecutively recruited in this prospective cohort study, and were accordingly divided into EPO group (*N* = 165) and ioversol group (*N* = 63). The inclusion criteria were as follows: 1) receiving HSG due to clinical condition and patients’ willingness; 2) aged 21–50 years old; 3) having no history of iodine allergy. The exclusion criteria were: 1) body temperature higher than 37.3 Centigrade within 3 days before the HSG; 2) known hyperthyroidism; 3) current uterine or cervical hemorrhage; 4) women who were in amenorrhea but were not ruled out for pregnancy; 5) severe heart or lung disease; 6) hematological malignancy. In addition, the raw data could be seen in the Additional file 1.

This study was approved by the Ethics Committee of Wuhan Third Hospital, Tongren Hospital of Wuhan University and conducted according to the Declaration of Helsinki. All patients signed informed consent before enrollment.

### Information collection

After enrollment, all the patients’ baseline characteristics of demography, clinical characteristics, medical history, complications and treatment history were recorded, which included age, body mass index (BMI), smoke, history of sex life, childbearing history, abortion history, duration of infertility, menstrual status, sexual behavior in menstruation, bath in menstruation, endocrine disorder, endometritis, previous chlamydia infection, pelvic inflammatory disease, coleitis, follicular estrogens, tuberculosis of uterus/tubal, cardio-cerebrovascular disease and previous surgery. Moreover, the sperm status of the patient’s male partner was recorded as well.

### HSG procedure

In this present prospective cohort study, all patients received oil-soluble contrast medium or water-based contrast medium for HSG according to disease condition and personal willingness. In terms of contrast medium used in the HSG procedure, those patients who received EPO Injection (Jiangsu Hengrui Medicine Co., Ltd. China) for HSG were allocated to EPO group (*N* = 165), and patients who received ioversol Injection (Jiangsu Hengrui Medicine Co., Ltd. China) for HSG were allocated into ioversol group (*N* = 63).

HSG was performed within 3–7 days after the patient’s menstruation was clean and sexual behavior was banned 3 days before the HSG, and bladder was emptied before the procedure. HSG was performed as follows: 1) the patient was placed in the supine position; 2) Routine disinfection was performed on the bladder lithotomy position of patient; 3) Vaginal speculum was used to expose the vagina and cervix uteri, then disinfection was also implemented; 4) A rubber double-lumen tube or a special catheter was inserted into cervix uteri, then was fixated; 5) Preheated EPO Injection or ioversol Injection was slowly infused into the uterus under appropriate pressure until adequate uterine filling occurred or contrast medium flew into pelvic cavity; 6) During the infusion, the dynamic flow of the course of the contrast medium in to the uterine cavity and fallopian tube was observed, and the films were taken before the contrast agent was injected and after the uterine cavity was filled with the fallopian tube development appeared; 7) When the film overlapped, the tube or bed position was changed if needed. A total of 6–8 ml EPO Injection or 8–10 ml ioversol Injection was given to complete the procedure, and for the patients who used the EPO Injection, the film was taken 24 h later, while for those patients who received ioversol Injection, the film was taken 20 min after infusion. After the procedure, films were reviewed by a radiologist, and a diagnosis was established.

### Assessment and follow up

After HSG, images were obtained and the quality of image was assessed according to the European Guidelines on quality criteria for diagnostic radiographic images [12] and scored by two radiologists independently as described in a previous study [13]. Assessment of the image quality consisted of 6 aspects including cervical canal display, shape and outline of uterus, cervical mucosa or folds, oviduct isthmus, the ampulla and fimbriae of oviduct and celiac diffuse image which were scored as follows: 0 (weakly visualized and not diagnostic), 1 (weakly visualized but diagnostic), 2 (good demonstration and diagnostic) and 3 (outstanding visualization), and total image quality score was the sum of the score of each aspect. Moreover, adverse events (AEs) during, within 2 h and 1-month post-HSG were recorded. Additionally, ultrasound sonography was used to evaluate the iodine residue at 1-month post-HSG.

### Statistics

Statistical analysis in this study was performed using SPSS 22.0 software (IBM Corp., Ltd., USA) and GraphPad 6.0 software (GraphPad Software Inc., USA). The required sample size for this study was calculated based on the estimation of 3 points difference in total image quality score between the two groups. A two-sided t test, 90% power were used to detect a difference in total image quality score of 3 points, with a two-sided 5% level of significance (α) and a sample size ratio of 1:2, required a sample size of 104 participants in EPO group and 52 in ioversol group. Considering that the attrition rate should be at least 15%, the sample size was inflated to 228 with 165 participants in EPO group and 63 in ioversol group. Data was presented as mean ± standard deviation, count (%) and median (25th–75th). Comparison between two groups was determined by t test, Wilcoxon rank sum test or Chi-square test. Univariate and multivariate linear regression analyses were performed to assess the independent value of EPO for predicting the image quality. *P* < 0.05 was considered significant.

## Results

### Baseline characteristics

As presented in Table 1, the mean age in EPO group and ioversol group were 31.36 ± 4.99 years and 31.40 ± 4.64 years, respectively (*P* = 0.960). The mean BMI values were 22.79 ± 6.11 kg/m^2^ and 21.97 ± 4.07 kg/m^2^, respectively (*P* = 0.319). Fourteen (8.5%) patients and 6 (9.5%) patients had a history of smoke in EPO group and ioversol group, respectively (*P* = 0.804). And the number of current smoker in two groups were 3 (1.8%) and 1 (1.6%) (*P* = 0.905). In addition, the median value of history of sex life was 7.0 (4.0–10.0) years and 7.0 (4.0–10.5) years in EPO group and ioversol group (*P* = 0.763). However, the percentage of patients had normal follicular estrogens was reduced but the proportions of patients had abnormal and unknown follicular estrogens were increased in EPO group compared with ioversol group (*P* = 0.009). Additionally, there was no difference regarding other baseline characteristics between the two groups (Table 1).

**Table 1:** Revised baseline characteristics of patients between EPO group and ioversol group

Parameters	EPO group (*N* = 165)	Ioversol group (*N* = 63)	*P* Value
Age (years)	31.36 ± 4.99	31.40 ± 4.64	0.960
BMI (kg/m^2^)	22.79 ± 6.11	21.97 ± 4.07	0.319
History of smoke (n/%)	14 (8.5)	6 (9.5)	0.804
Current smoker (n/%)	3 (1.8)	1 (1.6)	0.905
History of sex life (years)	7.0 (4.0–10.0)	7.0 (4.0–10.5)	0.763
Childbearing history			0.519
None (n/%)	106 (64.2)	36 (57.1)	
Once (n/%)	44 (26.7)	17 (27.0)	
Twice (n/%)	10 (6.1)	6 (9.5)	
≥ 3 times (n/%)	5 (3.0)	4 (6.4)	
Times of abortion			0.531
None (n/%)	78 (47.3)	30 (47.6)	
Once (n/%)	35 (21.2)	18 (28.6)	
Twice (n/%)	29 (17.6)	8 (12.7)	
≥ 3 times (n/%)	23 (13.9)	7 (11.1)	
The interval between last abortion and the present examination (months)	46.0 (23.0–81.0)	45.5 (12.0–63.3)	0.291
The way of last abortion			0.774
Accidental abortion (n/%)	3 (1.8)	3 (4.8)	
Induced by drugs (n/%)	19 (11.5)	5 (7.9)	
Artificial abortion (n/%)	60 (36.4)	22 (34.9)	
Painless induced abortion (n/%)	3 (1.8)	2 (3.2)	
Induced labour (n/%)	2 (1.2)	1 (1.6)	
Duration of infertility (years)	2.0 (1.0–4.0)	2.0 (1.5–4.0)	0.447
Spontaneous menstrual cycle (n/%)	149 (90.3)	59 (93.7)	0.427
Regular menstrual cycle (n/%)	140 (84.4)	56 (88.9)	0.432
Menstrual cycle (days)	29.0 (28.0–31.0)	30.0 (28.0–33.0)	0.088
Sexual behavior in menstruation			0.259
Never (n/%)	140 (84.8)	48 (76.2)	
Occasional (n/%)	20 (12.1)	13 (20.6)	
Often (n/%)	5 (3.0)	2 (3.2)	
Bath in menstruation			0.651
Never (n/%)	135 (81.8)	49 (77.8)	
Occasional (n/%)	26 (15.8)	13 (20.6)	
Often (n/%)	4 (2.4)	1 (1.6)	
Endocrine disorder (n/%)	12 (7.3)	3 (4.8)	0.700
Endometritis			0.923
Yes (n/%)	11 (6.7)	4 (6.3)	
No (n/%)	150 (90.9)	58 (92.1)	
Unknown (n/%)	4 (2.1)	1 (1.6)	
Previous chlamydia infection			0.209
Yes (n/%)	16 (9.7)	3 (4.8)	
No (n/%)	145 (87.9)	60 (95.2)	
Unknown (n/%)	4 (2.4)	0 (0.0)	
Pelvic inflammatory disease			0.370
Yes (n/%)	86 (52.1)	33 (52.4)	
No (n/%)	74 (44.8)	30 (47.6)	
Unknown (n/%)	5 (3.0)	0 (0.0)	
Coleitis			0.258
Yes (n/%)	87 (52.7)	39 (61.9)	
No (n/%)	74 (44.8)	24 (38.1)	
Unknown (n/%)	4 (2.4)	0 (0.0)	
Follicular estrogens			**0.009**
Normal (n/%)	135 (81.8)	61 (96.8)	
Abnormal (n/%)	11 (6.7)	2 (3.2)	
Unknown (n/%)	19 (11.5)	0 (0.0)	
Tuberculosis of uterus/tubal			0.650
Yes (n/%)	5 (3.0)	1 (1.6)	
No (n/%)	159 (96.4)	61 (96.8)	
Unknown (n/%)	1 (0.6)	1 (1.6)	
Previous surgery			0.673
Hysteroscopy (n/%)	12 (7.3)	8 (12.7)	
Laparoscope (n/%)	12 (7.3)	4 (6.3)	
Cesarean section (n/%)	12 (7.3)	2 (3.2)	
Tubal surgery (n/%)	6 (3.6)	2 (3.2)	
Ectopic pregnancy surgery (n/%)	6 (3.6)	1 (1.6)	
Others (n/%)	30 (18.2)	8 (12.7)	

Data was presented as mean value±standard deviation, median (quartile 25th–75th) or count (%). Comparison was determined by t test, Wilcoxon rank sum test or Chi-square test

*EPO* ethiodized poppyseed oil, *BMI* body mass index

*P* value in boldface stood for statistical significant

### Comparison of image quality between EPO group and ioversol group

**Fig. 1:**
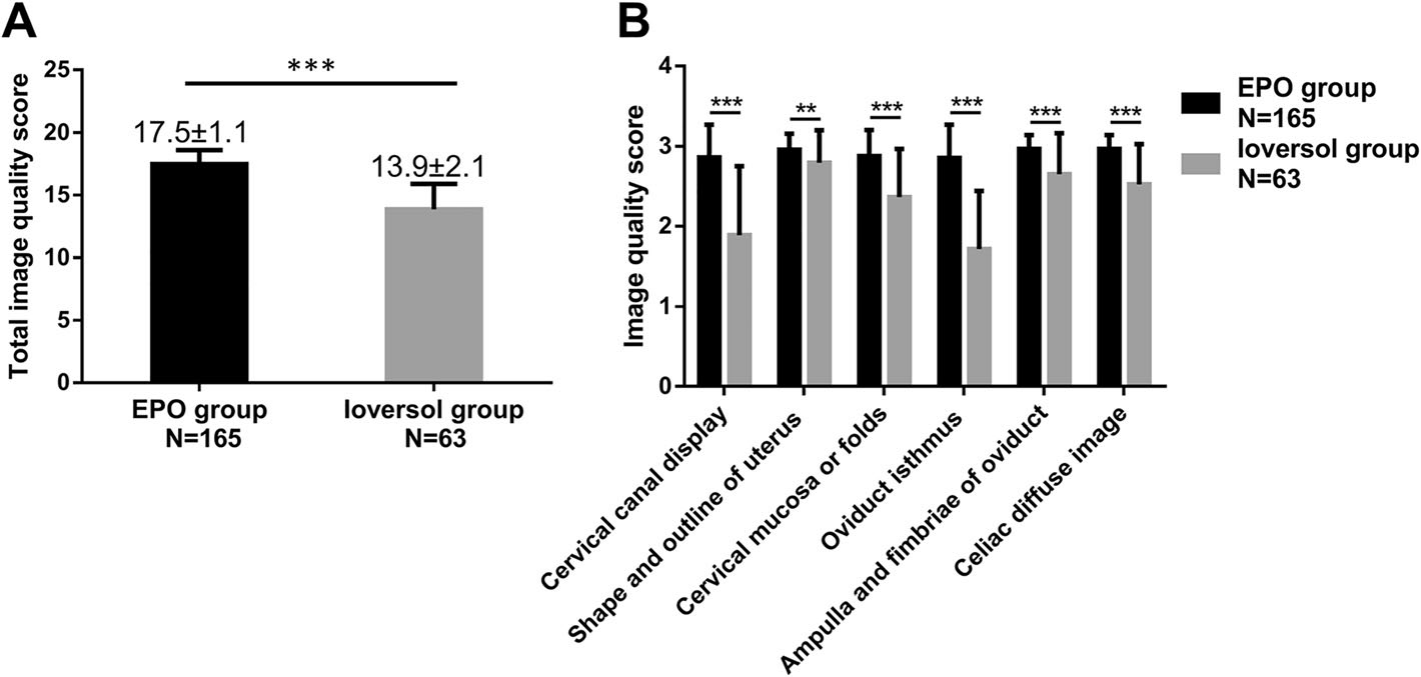
Comparison of image quality in EPO group and ioversol group. The mean total score was increased in EPO group compared with ioversol group (**a**), and the other detailed scores, including cervical canal display score, shape and outline of uterus score, cervical mucosa or folds score, oviduct isthmus score, ampulla and fimbriae of oviduct score and celiac diffuse image score were also higher than that in ioversol group (**b**). Comparison between two groups was determined by t test. *P* < 0.05 was considered significant. EPO, ethiodized poppyseed oil

**Fig. 2:**
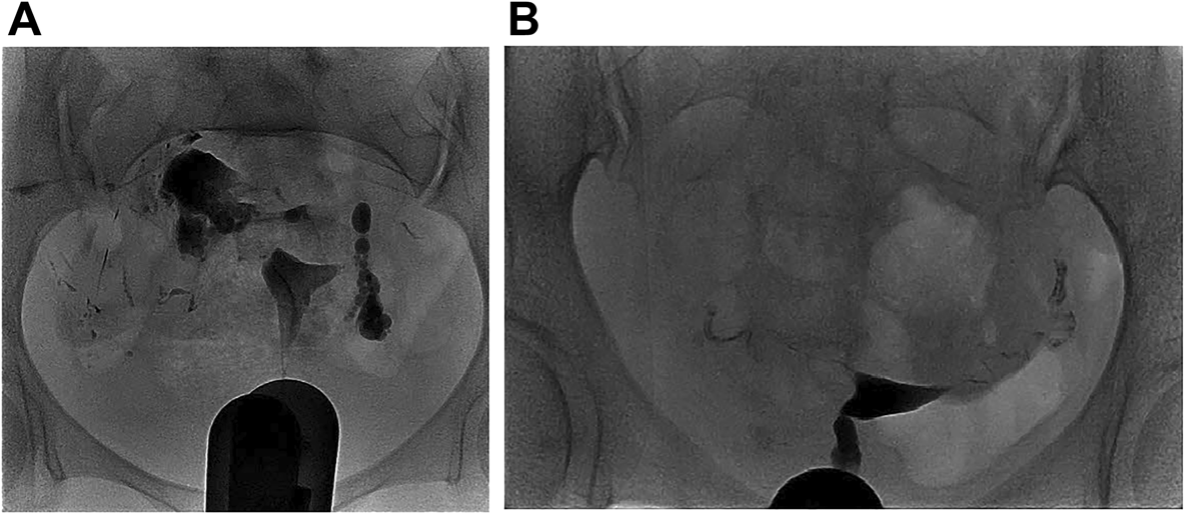
Hysterosalpingograms in the EPO group and ioversol group. The examples of hysterosalpingogram of patients in the EPO group (**a**) and ioversol group (**b**). EPO, ethiodized poppyseed oil

As shown in Fig. 1a, the mean total score in EPO group was elevated compared with ioversol group (*P* < 0.001). For the scores of detailed aspects, the cervical canal display score (*P* < 0.001), shape and outline of uterus score (*P* < 0.01), cervical mucosa or folds score (*P* < 0.001), oviduct isthmus score (*P* < 0.001), ampulla and fimbriae of oviduct score (*P* < 0.001) and celiac diffuse image score (*P* < 0.001) were all increased in EPO group than those in ioversol group (Fig. 1b). In addition, examples of hysterosalpingograms in EPO group (Fig. 2a) and in ioversol group (Fig. 2b) were presented as well.

### Factors affecting total image quality score

To assess the independent predictive value of EPO for total image quality score, the univariate linear regression and multivariate linear regression were conducted. As presented in Table 2, univariate linear regression revealed that EPO (*P* < 0.001) was positively correlated with the total image quality score. Furthermore, all factors were included in the multivariate linear regression, which showed that EPO (*P* < 0.001) was an independent predictive factor for increased total image quality score.

**Table 2:** Factors affecting the total image quality score

	Univariate linear regression				Multivariate linear regression			
Parameters	*P* value	B	95% CI		*P* value	B	95% CI	
			Lower	Higher			Lower	Higher
EPO vs ioversol	**< 0.001**	3.640	3.222	4.058	**< 0.001**	3.805	2.997	4.613
Age	0.981	−0.001	−0.060	0.058	0.061	0.115	− 0.005	0.235
BMI	0.828	−0.006	− 0.057	0.046	0.828	−0.009	− 0.094	0.075
History of smoke	0.815	0.119	− 0.883	1.121	0.975	−0.025	−1.573	1.523
Current smoker	0.349	1.027	−1.129	3.182	0.472	0.851	−1.507	3.209
History of sex life	0.555	0.018	−0.042	0.079	0.134	−0.107	− 0.249	0.034
Times of Childbearing	0.514	−0.119	−0.477	0.239	0.667	−0.098	−0.552	0.356
Times of abortion	0.421	0.082	−0.119	0.284	0.753	0.066	−0.352	0.484
The interval between last abortion and the present examination	0.360	0.004	−0.005	0.012	0.370	0.006	−0.007	0.019
Abortion	0.558	0.169	−0.399	0.738	–	–	–	–
Duration of infertility	0.794	0.005	−0.030	0.039	0.284	− 0.093	− 0.267	0.080
Spontaneous menstrual cycle	0.794	0.005	−0.030	0.039	0.354	0.637	−0.729	2.002
Regular menstrual cycle	0.582	0.228	−0.587	1.044	0.828	−0.133	−1.353	1.088
Menstrual cycle	0.434	−0.023	−0.082	0.036	0.958	−0.003	−0.111	0.106
Sexual behavior in menstruation	0.365	−0.275	−0.871	0.322	0.878	0.094	−1.133	1.322
Bath in menstruation	0.647	0.143	−0.472	0.758	0.410	0.368	−0.520	1.255
Endocrine disorder	0.489	0.402	−0.741	1.544	0.306	0.836	−0.788	2.461
Endometritis	0.499	0.391	−0.748	1.529	0.566	−0.397	−1.776	0.982
Previous chlamydia infection	0.159	0.738	−0.292	1.768	0.719	−0.243	−1.592	1.106
Pelvic inflammatory disease	0.898	0.037	−0.527	0.600	0.172	0.588	−0.264	1.440
Coleitis	0.948	−0.019	−0.599	0.561	0.800	−0.107	−0.951	0.737
Follicular estrogens abnormal	0.529	0.402	−0.854	1.658	0.555	0.509	−1.211	2.229
Tuberculosis of uterus/tubal	0.435	−0.694	−2.444	1.056	0.201	2.141	−1.176	5.458
Previous surgery	0.170	0.395	−0.171	0.960	0.710	−0.153	−0.971	0.666
Sperm of male partner (abnormal)	0.468	−0.282	−1.047	0.483	0.742	0.157	−0.791	1.105

Data was presented as *P* value, B (Unstandardized Coefficient) and 95% CI (confidence interval). Univariate and multivariate linear regression was conducted to determine the factors affecting the total image quality score

*EPO* ethiodized poppyseed oil, *BMI* body mass index

*P* value in boldface stood for statistical significant

### AEs during operation

The numbers of patients with mild, moderate and severe abdominal pain (*P* = 0.134), nausea (*P* = 0.807), emesis (*P* = 0.218), colporrhagia (*P* = 0.217) and lymphatic reflux (*P* = 1.000) during operation were similar between EPO group and ioversol group (Table 3). In addition, there was no patient presented with allergy or venous reflux during the operation in either group.

**Table 3:** Adverse events during operation

Parameters	EPO group (*N* = 165)	Ioversol group (*N* = 63)	*P* Value
Abdominal pain			0.134
Mild (n/%)	61 (27.0)	32 (50.8)	
Moderate (n/%)	81 (49.1)	27 (42.9)	
Severe (n/%)	13 (7.9)	1 (1.6)	
Nausea (n/%)	23 (13.9)	8 (12.7)	0.807
Emesis (n/%)	7 (4.2)	0 (0.0)	0.218
Allergy (n/%)	0 (0.0)	0 (0.0)	–
enous reflux (n/%)	0 (0.0)	0 (0.0)	–
Colporrhagia (n/%)	23 (13.9)	5 (7.9)	0.217
Lymphatic reflux (n/%)	1 (0.6)	0 (0.0)	1.000

Data was presented as count (%). Comparison was determined by Chi-square test

*EPO*, ethiodized poppyseed oil

*P* Value < 0.05 was considered statistically significant

### AEs within 2 h post operation

As listed in Table 4, there were also no differences regarding the occurrences of mild, moderate or severe abdominal pain (*P* = 0.433), back pain (*P* = 0.752), shoulder pain (*P* = 0.955), nausea (*P* = 0.259), emesis (*P* = 0.593), allergy (*P* = 0.593) or colporrhagia (*P* = 0.371) within 2 h post operation between EPO group and ioversol group.

**Table 4:** Adverse events occurred within 2 h post operation

Parameters	EPO group (*N* = 165)	Ioversol group (*N* = 63)	*P* Value
Abdominal pain			0.433
Mild (n/%)	80 (48.5)	34 (54.0)	
Moderate (n/%)	22 (13.3)	6 (9.5)	
Severe (n/%)	5 (3.0)	0 (0.0)	
Back pain (n/%)	4 (2.4)	2 (3.2)	0.752
Shoulder pain (n/%)	5 (3.0)	2 (3.2)	0.955
Nausea (n/%)	22 (13.3)	5 (7.9)	0.259
Emesis (n/%)	13 (7.9)	3 (4.8)	0.593
Allergy (n/%)	1 (0.6)	0 (0.0)	0.536
Colporrhagia (n/%)	23 (13.9)	6 (9.5)	0.371

Data was presented as count (%). Comparison was determined by Chi-square test

*EPO* ethiodized poppyseed oil

*P* Value < 0.05 was considered statistically significant

### AEs at 1-month post operation

However, at 1st month after operation, the number of patients with unchanged, deepened and faded menstrual blood color in EPO group were different from ioversol group (*P* = 0.007) (Table 5). The proportions of patients had unchanged menstrual color (92.1% vs 98.8%, respectively) and faded menstrual color (0.0% vs 0.6%, respectively) were elevated but the percentage of patients had deepened menstrual color (7.9% vs 0.6%, respectively) was reduced in EPO group compared with ioversol group. In addition, the number of patients had iodine residue in uterine cavity in EPO group was increased compared with ioversol group (*P* < 0.001). The incidences of dysmenorrhea (*P* = 0.794) and amount of menstruation (*P* = 0.254) were similar between the two groups.

**Table 5:** Assessment at 1st month after operation

Parameters	EPO group (*N* = 165)	Ioversol group (*N* = 63)	*P* Value
Dysmenorrhea			0.794
Disappear (n/%)	1 (0.6)	1 (1.6)	
Unchanged (n/%)	159 (96.4)	61 (96.8)	
Alleviated (n/%)	4 (2.4)	1 (1.6)	
Aggravated (n/%)	1 (0.6)	0 (0.0)	
Amount of menstruation			0.254
Unchanged (n/%)	161 (97.6)	59 (53.7)	
Decreased (n/%)	2 (1.2)	1 (1.6)	
Increased (n/%)	2 (1.2)	3 (4.8)	
Menstrual blood color			**0.007**
Unchanged (n/%)	163 (98.8)	58 (92.1)	
Deepened (n/%)	1 (0.6)	5 (7.9)	
Faded (n/%)	1 (0.6)	0 (0.0)	
Iodine residue in uterine cavity (n/%)	36 (21.8)	0 (0.0)	**< 0.001**

Data was presented as count (%). Comparison was determined by Chi-square test

*EPO* ethiodized poppyseed oil

*P* value in boldface stood for statistical significant

## Discussion

In this study, we found that (1) the total image quality score, cervical canal display score, shape and outline of uterus score, cervical mucosa or folds score, oviduct isthmus score, ampulla and fimbriae of oviduct score as well as celiac diffuse image score were all increased in EPO group compared with ioversol group; (2) there was no difference of AEs during and within 2 h post operation between EPO group and ioversol group. However, at 1-month post HSG, the percentages of patients had unchanged menstrual blood color and faded menstrual blood color, which was detected by ultrasound sonography, increased while the rate of patients with deepened menstrual blood color was reduced in EPO group compared to ioversol group. And the number of patients with iodine residue in uterine cavity was increased in EPO group compared with ioversol group.

Oil-based contrast agents and water-soluble contrast agents have been compared by various clinical studies and clinical trials for over a decade, and the aims of those studies mostly focus on the thyroid function and fertility rate post-HSG. A previous multicenter, randomized clinical trial in the Netherlands elucidates that rates of ongoing pregnancy and live birth in patients receiving HSG using an oil-based contrast agent are both higher compared with a water-soluble contrast agent [10]. An earlier randomized, controlled trial includes proven tubal obstruction patients treated by HSG using oil-based contrast agent as oil group and the patients receiving no intervention as control group, and their results illuminate that the accumulating rates of pregnancy at 18 months are similar but the meantime before achieving pregnancy is shorter in the oil group compared to control group [14]. The oil contrast used in our study was EPO, which has been used for HSG for a long time and is demonstrated to be superior to water-soluble contrast agent in enhancing the fertility rate in women post-HSG as early as in the 1990s [15, 16]. However, to the best of our knowledge, there is still no study comparing the image quality of oil-based contrast agents compared to water-soluble contrast agents in HSG. In our study, we found that the scores of image quality assessed by the European Guidelines on quality criteria for diagnostic radiographic images in EPO group were higher than those in ioversol group. The probable explanations of our results are as follows (1) the time duration between contrast agent injection and the beginning of picturing was 24 h in EPO group, while was only 20 mins in ioversol group, suggesting that physicians in the EPO group had much more time to observe and take pictures, which might somehow ensure a better quality of images. (2) According to previous studies, the effect of oil contrast agent on removing or flushing the mucus plug residue remained in the fallopian tubes is better than the water-soluble contrast, which ensures a more clear image during HSG [10, 17].

Due to the allergic reaction, iodine residue and influenced thyroid function, which is occasionally observed in practice and studies, the short-term and long-term safety profile of iodine-based contrast agent requires for attention [10, 18, 19]. However, severe AEs are rare, such as pulmonary and cerebral oil embolism, are rare and are not observed in the majority of previous studies [10, 18]. In our study, the incidence of AEs during and within 2 h post-HSG in EPO group and ioversol group are similar, indicating EPO and ioversol were both tolerable during and post operation. In the study of Kim Dreyer et al., the incidence of AEs are low in both oil-based contrast agent group and water-soluble contrast agent group, and they discover no difference of the incidence of AEs between two groups, which is partly in accordance with ours [10]. In addition, at 1-month post-HSG, the rates of patients with unchanged and faded menstrual blood color was higher, and the percentage of patients with deepened menstrual blood color was decreased in EPO group compared with ioversol group. These results suggested that the color change of menstrual blood was less severe in the EPO group compared with the ioversol group, which may also indicate that the menstruation might be less influenced by HSG using EPO as contrast agent. With respect to the causes of the changed menstrual blood color in the two groups, it could be explained by that (1) in the EPO group, the color change of menstrual blood might be caused by the iodine residue in the uterine cavity; (2) while in the ioversol group, it may be caused by some other reasons or chance. The proportion of patients with iodine residue in uterine cavity which was assessed by ultrasound sonography at 1-month post-HSG was elevated in EPO group compared with ioversol group, and no patient in ioversol group was found to have iodine residue in our study. Ioversol is a water-soluble contrast agent that leaves no iodine residue in patients. However, it is normal for the oil-based contrast agent EPO to cause iodine residue in the uterine cavity due to EPO had a worse fluidity compared with ioversol, which had an excellent fluidity as a water-soluble agent.

### Limitations

There were several limitations in our study. (1) As a cohort study, there were some bias and confounding factors in our study. For example, the proportion of patients with distinct follicular estrogens levels at baseline were different between the two groups, which was a confounding factor that might have an influence on our results. However, we conducted multivariate linear regression, which showed that the level of follicular estrogens has no influence on the results of our study. (2) This was a single center study only recruited patients from central China, which caused selection bias. (3) The sample size was small. (4) The two radiologists responsible for image quality evaluation were not blinded to the contrast that was used in the procedure, which may cause observer bias; (5) the randomization was not performed in this study. Thus, a multicenter, double-blind, randomized clinical trial with enlarged sample size is needed in the future.

## Conclusions

In conclusion, EPO is more efficient in image quality and equally tolerant compared to ioversol as contrast agents in HSG.

## Additional file

Additional file 1: RAW DATA. (XLS 157 kb)

## Data Availability

Available with this manuscript.

## Abbreviations

AEs: Adverse events
BMI: Body mass index
CT: Computerized tomography
EPO: Ethiodized poppyseed oil
HSG: Hysterosalpingography
